# ARISE framework: a temporal, multi-organ view of perfusion in sepsis

**DOI:** 10.1007/s11739-026-04441-8

**Published:** 2026-06-22

**Authors:** Jingjing Liu, Hua Zhao, Dawei Liu, Xiaoting Wang

**Affiliations:** Department of Critical Care Medicine, Peking Union Medical College Hospital, Peking Union Medical College, Chinese Academy of Medical Sciences, 1# Shuai Fu Yuan, Dong Cheng District, Beijing, 100730 China

**Keywords:** Sepsis, Redistribution of blood flow, Arterial resistance index, ARISE, Ultrasound

## Abstract

Sepsis is a critical illness characterized by pronounced temporal dynamics and marked heterogeneity of organ perfusion. Current hemodynamic management relies largely on global macro-circulatory variables such as MAP and CO, but these measures provide limited insight into inter-organ blood-flow redistribution and often fail to detect occult hypoperfusion in vulnerable tissues. Consequently, organ dysfunction may continue to progress even when systemic targets appear to be achieved. The arterial resistance index (RI) can be repeatedly assessed in specific organs with relatively high temporal resolution, it provides a practical signal for tracking perfusion heterogeneity in sepsis. On this basis, this review reframes sepsis as a process dominated by dynamic blood-flow redistribution rather than a single, homogeneous state of circulatory failure and proposes the Arterial Resistance Index Series in Echography (ARISE) framework. ARISE focuses on four representative vascular beds that capture key dimensions of circulatory regulation—including central control, vulnerable organs, and peripheral perfusion: the cerebral, renal, and superior mesenteric arteries, as well as the anatomical snuffbox artery. The framework emphasizes RI trajectories over time, inter-organ flow distribution, and the structured integration of these patterns. Available evidence indicates that arterial RI in different organs exhibits pronounced temporal variation and inter-organ heterogeneity during sepsis. Distinct trajectories are observed across central, vulnerable, and peripheral vascular beds, reflecting ongoing redistribution of blood flow as the disease evolves. Multi-organ RI assessment can reveal pathophysiological phenomena not captured by conventional macro-circulatory indices, including occult hypoperfusion, macro-microcirculatory uncoupling, and “sacrificial” redistribution. Advances in critical care ultrasonography now enable bedside, real-time, quantitative monitoring of multi-organ RI, providing the technical foundation for dynamic perfusion assessment. Overall, ARISE shifts sepsis assessment from a static pressure-flow paradigm to a dynamic framework centered on organ blood-flow distribution and evolving perfusion patterns.

## Background

Sepsis3.0 defines sepsis as life‑threatening organ dysfunction caused by a dysregulated host response to infection. Current therapeutic strategies for sepsis primarily focus on rapid source control, stabilization of macro-circulatory hemodynamics, and support of organ function [1]. With ongoing advances in antimicrobial therapy, vasoactive agents, and life‑support technologies, source control and global hemodynamic targets—such as mean arterial pressure (MAP) and cardiac output(CO)—can often be achieved within a relatively short time frame [2–4].However, accumulating evidence indicates that organ‑level hypoperfusion may persist despite normalization of systemic hemodynamic parameters, suggesting that reliance on traditional macro-circulatory indices alone is insufficient to accurately reflect true organ perfusion status [5–7]. In addition, substantial inter‑organ heterogeneity in perfusion exists, along with dynamic evolution of this heterogeneity over the course of the disease. Consequently, how to dynamically and precisely assess organ perfusion at the bedside has become a central challenge in the hemodynamic management of sepsis.

Organ blood flow is primarily determined by CO [8], organ perfusion pressure [9], and organ‑specific vascular resistance [10]. Under contemporary critical care conditions, CO can usually be effectively maintained through pharmacologic or mechanical support. Organ perfusion pressure, in turn, is closely related to MAP and organ‑specific vascular resistance [11]. When MAP is relatively stable, vascular resistance becomes the key determinant of organ perfusion and blood‑flow distribution, directly governing inter‑organ blood‑flow redistribution and its temporal dynamics during sepsis [12]. Given the substantial differences among organs in anatomical structure, vascular regulatory mechanisms, and sensitivity to inflammatory mediators, organ perfusion during sepsis is characterized by marked heterogeneity and dynamic variability—features that are often not captured by single, global hemodynamic metrics.

Against this background, bedside ultrasonography has increasingly emerged as an important tool for organ‑level hemodynamic assessment. Critical care ultrasound enables noninvasive, real‑time evaluation of both macro-circulatory status and organ‑specific perfusion through Doppler measurements across multiple vascular beds. Among Doppler-derived indices, the arterial resistance index (RI), usually calculated as (peak systolic velocity − end-diastolic velocity) / peak systolic velocity, is widely used as a surrogate descriptor of downstream vascular impedance and perfusion conditions. Importantly, RI is regarded as an integrated signal influenced by arterial compliance, distal resistance, pulse pressure transmission, and downstream perfusion pressure. Nevertheless, RI offers practical advantages including bedside feasibility, broad availability, and suitability for repeated dynamic monitoring. These characteristics make RI particularly attractive in sepsis, a condition defined by rapid progression, pronounced inter-organ heterogeneity, and substantial blood-flow redistribution.

Nevertheless, previous studies have largely interpreted RI as a local marker of single‑organ perfusion, with limited integration of RI patterns across different organs or exploration of their interrelationships. In addition, prior work has often attempted to define single cutoff values for abnormal RI, but these thresholds vary substantially across vascular beds, populations, disease stages, and treatment contexts, thereby limiting their generalizability. In sepsis—where blood‑flow redistribution and reprioritization of perfusion represent core pathophysiological features—isolated assessment of individual organ parameters may fail to fully capture the global perfusion strategy and its evolution over time. There is therefore a clear need for an evaluative framework capable of integrating multi‑organ perfusion information and distinguishing adaptive from maladaptive redistribution states.

On this basis, we introduce the ARISE (Arterial Resistance Index Series in Echography) framework, which uses ultrasound‑derived arterial resistance indices as central functional markers to systematically integrate perfusion information from multiple organ vascular beds. This review focuses on the temporal evolution of arterial RI in different organs during sepsis and their interrelationships and explores their associations with organ dysfunction. Our aim is to elucidate the potential value of RI in characterizing blood‑flow redistribution, perfusion heterogeneity, and continuous assessment in sepsis, thereby providing a novel physiological foundation for organ‑oriented dynamic evaluation and individualized management strategies. However, at present, ARISE should still be regarded as a conceptual, hypothesis-generating framework rather than a validated and standardized protocol ready for routine clinical application. To address this gap, our research team is currently conducting a clinical study in patients with sepsis to evaluate the predictive value of ARISE for important clinical outcomes, including organ dysfunction, the need for continuous renal replacement therapy (CRRT), duration of vasoactive agent use, and 28-day mortality, with the aim of providing practical evidence and feasible implementation strategies for the ARISE framework.

## Literature search strategy and scope of the review

This review is a narrative, physiology-oriented synthesis supported by a structured literature search. We searched PubMed, Web of Science, and Embase for studies published in English up to, using combinations of the following keywords: “sepsis,” “septic shock,” “resistance index,” “resistive index,” “Doppler ultrasound,” “renal resistance index,” “cerebral blood flow,” “mesenteric perfusion,” “microcirculation,” and “point-of-care ultrasound.” Original clinical studies, relevant experimental studies, and high-quality review articles were considered when they contributed to the physiological interpretation or bedside applicability of arterial resistance index (RI) assessment in sepsis. Particular attention was paid to studies addressing organ-specific perfusion, Doppler-derived vascular resistance, serial hemodynamic assessment, and sepsis-associated organ dysfunction. Studies not directly related to sepsis, organ perfusion, Doppler-derived RI, or bedside hemodynamic monitoring were not included in the conceptual synthesis. Given the heterogeneity of the available evidence and the conceptual aim of the manuscript, this review does not constitute a formal systematic review or meta-analysis; instead, it provides an integrative framework to organize current evidence and identify major knowledge gaps.

## From disordered blood-flow redistribution to bedside signals: the rationale for the ARISE framework

Circulatory failure during sepsis is manifested not only as global hemodynamic derangement but, more importantly, as profound disruption of regional blood‑flow distribution. Increasing evidence suggests that the development of sepsis‑associated multiple organ dysfunction syndrome (MODS) is driven less by an absolute deficit in total blood flow than by maladaptive redistribution of blood flow between and within organs, even when systemic circulatory parameters appear to have been corrected [7, 13, 14].

At the macro-circulatory level, inflammation‑driven dysregulation of vascular tone represents the initiating event in blood‑flow redistribution. Excessive release of pro‑inflammatory cytokines and nitric oxide induces systemic vasodilation [13, 15]. However, marked heterogeneity in the responsiveness of different vascular beds to inflammatory mediators results in disproportionate dilation of low‑resistance territories. In contrast, perfusion to organs with tightly regulated autoregulation (e.g., the kidneys and brain) may become relatively constrained, leading to inter‑organ perfusion imbalance [16]. Although neurohumoral mechanisms can provide early compensation by promoting “centralization” of blood flow, their regulatory capacity progressively wanes with persistent inflammation and worsening vascular hyperresponsiveness [17]. Consequently, regional perfusion patterns in later stages of the disease often become unpredictable and potentially injurious.

Concurrently, microcirculatory dysfunction further amplifies the consequences of macro-circulatory imbalance. Endothelial injury, glycocalyx degradation, and microvascular obstruction reduce the density of effectively perfused capillaries, giving rise to functional shunting and a dissociation between macro-circulatory blood flow and tissue oxygen utilization—so‑called macro–microcirculatory uncoupling [18, 19]. Abnormal hemorheology and microthrombus formation preferentially compromise distal micro-vessels, exacerbating inter‑ and intra‑organ perfusion heterogeneity and magnifying tissue hypoxia at the cellular level [20, 21].

Beyond vascular mechanisms, alterations in organ metabolic demand also profoundly shape blood‑flow distribution. In sepsis, immune activation becomes a highly energy‑consuming process, whereas parenchymal organs may actively downregulate metabolic requirements in response to mitochondrial dysfunction, entering a state akin to “tolerant hypoperfusion” or functional hibernation [22–24]. In this context, blood‑flow redistribution is not merely a passive consequence of circulatory derangement but may also represent an adaptive reconfiguration across perfusion status, microcirculatory integrity, and metabolic demand [25, 26].

Under the regulation of the mechanisms described above, vascular resistance varies across different organs. This heterogeneity drives redistribution of blood flow toward low‑resistance territories, with perfusion preferentially directed to organs with lower vascular resistance. As a result, inter‑organ perfusion mismatch may occur even when CO is preserved or increased. Accordingly, sepsis‑associated organ dysfunction cannot be explained by abnormalities in any single flow parameter, rather, it represents a dynamic disequilibrium spanning the macro-circulatory, microcirculatory, and metabolic domains. Reliance on conventional macro-circulatory targets alone is therefore insufficient to meet the demands of organ protection and individualized therapy, highlighting the need for an assessment tool capable of repeatedly and non‑invasively capturing organ‑specific perfusion signals and their dynamic changes at the bedside.

Bedside Doppler ultrasonography provides real‑time characterization of vascular resistance, is accessible across multiple organs, and does not rely on assumptions of global hemodynamic stability, thereby serving as an important interface between macro-circulatory status and regional perfusion regulation. Existing studies have proposed widely varying thresholds for defining an “abnormal” RI, and its diagnostic and prognostic performance is often substantially influenced by small sample sizes, heterogeneity of study populations, inconsistent acquisition protocols, and important confounding factors such as age, vascular compliance, venous congestion, vasoactive support, and depth of sedation [27]. In addition, RI is a dynamic hemodynamic parameter determined by both peak systolic velocity and end-diastolic velocity and is therefore highly susceptible to changes in cardiac output, pulse pressure, volume status, mechanical ventilation, intra-abdominal pressure, and therapeutic interventions. Comparability across studies is further limited by differences in the vascular beds examined, sampling sites, timing of measurements, outcome definitions, and statistical approaches used to derive cutoff values. In the highly dynamic pathophysiological context of sepsis, however, single measurements of the RI are insufficient to distinguish compensatory from maladaptive blood‑flow regulation and cannot reflect the directional evolution of organ perfusion in response to disease progression or therapeutic interventions. Consequently, serial and dynamic assessment of RI, rather than isolated interpretation of single measurements, is essential for understanding the process of blood‑flow redistribution.

On this basis, we propose the ARISE framework, which integrates dynamic series of Doppler‑derived RI measurements obtained at the bedside to elucidate dysregulated blood‑flow control and its organ‑specific manifestations in sepsis. The ARISE framework is built upon the following core elements:

- Multi‑organ orientation: ARISE framework integrates RI signals derived from multiple organs to capture both inter‑organ and intra‑organ heterogeneity of blood‑flow regulation in sepsis, rather than assuming uniform systemic perfusion changes.
- Serial assessment: The framework emphasizes repeated and continuous RI measurements to characterize the temporal evolution of circulatory regulatory states, rather than relying on isolated static values.
- Trend‑ and divergence‑based interpretation: Particular attention is paid to the direction and magnitude of RI changes, as well as divergence in trajectories among different organs, to identify potential patterns of blood‑flow redistribution. “divergence” refers to the non-parallel evolution of RI across different vascular beds. For example, after early resuscitation for septic shock, a patient may exhibit a decrease in lactate levels, shortened capillary refill time, warming of the extremities, and a reduction in peripheral arterial RI compared with baseline, suggesting improvement in global hemodynamics and peripheral perfusion. However, renal arterial or superior mesenteric arterial RI may remain persistently elevated or even worsen relative to the initial measurement. Similarly, in some patients receiving norepinephrine support, MAP may be restored to the target range of 65–75 mmHg, yet urine output may remain reduced, bowel sounds diminished, or abdominal distension worsened. In this context, if renal or mesenteric RI does not decrease in parallel with MAP improvement but instead continues to increase, it suggests that “pressure restoration” does not necessarily equate to “perfusion restoration.” Reliance on MAP targets alone may therefore obscure ongoing organ-level ischemic risk.
- Non‑threshold‑based strategy: Fixed RI cutoff values are deliberately avoided. Instead, RI dynamics are interpreted within the broader clinical context of the individual patient, thereby mitigating the influence of inter‑individual variability and measurement‑related factors.

**Fig. 1:**
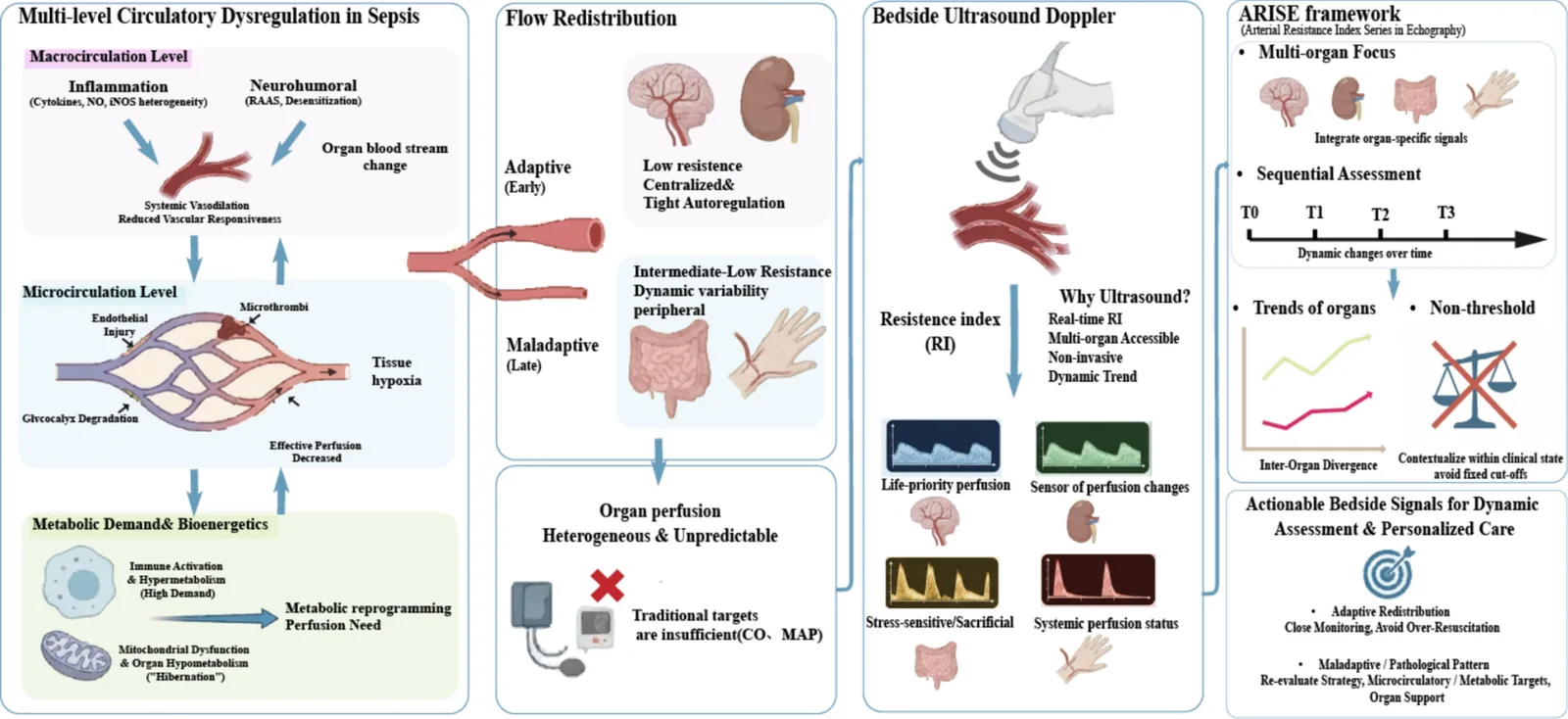
From multi-level dysregulation to bedside signals: the ARlSE framework for sepsis

Through these principles, ARISE framework translates multilevel circulatory dysregulation into bedside‑accessible and longitudinally traceable imaging signals, providing a pragmatic clinical framework for the dynamic assessment of blood‑flow redistribution in sepsis, while acknowledging that this approach still requires prospective validation and standardization before routine adoption, as Fig. 1.

Imbalance develops across multiple interconnected levels in sepsis: (1) inflammatory and neuroendocrine responses at the macro-circulatory level led to the coexistence of systemic vasodilation and blood-flow redistribution; (2) at the microcirculatory level, endothelial injury and micro-thrombosis reduce effective perfusion and result in macro–microcirculatory uncoupling; and (3) immune activation and organ-specific shifts in metabolic demand further interact with these processes. Collectively, these interactions culminate in inter-organ redistribution of blood flow. Conventional monitoring based on cardiac output (CO) and mean arterial pressure (MAP) cannot reliably capture true organ perfusion. In contrast, bedside Doppler ultrasound enables noninvasive and repeatable acquisition of multi-organ arterial resistive indices (RI), translating the above pathophysiology into actionable perfusion signals. ARISE (Arterial Resistance Index Series in Echography) emphasizes moving from a single RI “snapshot” to trend interpretation using serial measurements (T1–T3). By integrating multi-organ RI trajectories to identify patterns of inter-organ perfusion redistribution, ARISE provides dynamic information to support individualized hemodynamic management.

## Organ-specific interpretation of resistance index within the ARISE framework

ARISE framework emphasizes cross‑organ comparison of RI trajectories on a unified time axis, rather than single‑organ interpretation. This requires defining the organ‑specific physiological meaning and regulatory basis of RI in sepsis. Given differences in autoregulation, microvascular architecture, and metabolic demand across the cerebral, renal, mesenteric, and peripheral arteries, we will summarize the pathophysiological rationale and clinical evidence for RI in each bed and integrate these findings across central, vulnerable, and peripheral organs.

### Cerebral resistance index: preserved autoregulation, centralization, and late failure

As the organ with the highest metabolic demand and the lowest tolerance to hypoxia, the cerebral arterial occupies a unique position in sepsis‑related blood‑flow redistribution. It is both the primary target of early circulatory “centralization” and the site where failure of this protective mechanism leads to the most devastating consequences. Understanding cerebral arterial RI therefore requires insight into its physiological regulatory basis and how this regulation collapses under inflammatory stress.

#### Physiological basis of cerebral blood flow autoregulation and disruption

The brain lacks meaningful reserves of glucose and oxygen, and maintenance of its function is therefore highly dependent on continuous and stable cerebral blood flow (CBF). Under physiological conditions, the cerebral vascular bed is equipped with highly refined autoregulatory mechanisms.

First, pressure autoregulation ensures that when MAP fluctuates within a certain range (typically 60–140 mmHg), cerebral arterioles constrict or dilate via myogenic mechanisms to maintain relatively constant CBF [28, 29]. This pressure–flow plateau indicates an active, positive regulation of cerebrovascular resistance in response to changes in perfusion pressure within this range. Second, metabolic autoregulation renders cerebral vessels exquisitely sensitive to changes in arterial PaCO₂ [30, 31]. An increase in PaCO₂ induces marked vasodilation and a reduction in vascular resistance, whereas a decrease leads to vasoconstriction [32].

In sepsis, however, this finely tuned regulation is frequently disrupted. Systemic inflammation, endothelial dysfunction, and imbalanced vascular smooth‑muscle responsiveness to vasoconstrictors (e.g. endothelin) and vasodilators (e.g. NO) collectively impair the ability of cerebral arteries to regulate vascular tone [33, 34].

#### Cerebral RI changes during sepsis: from compensation to maladaptation

During septic shock, profound systemic vasodilation leads to arterial hypotension. In response, neurohumoral mechanisms are activated to increase peripheral vascular resistance and “centralize” blood flow toward vital organs, particularly the brain and heart. At this stage, if cerebral autoregulation is preserved, cerebral arterioles are expected to dilate to maintain perfusion, theoretically resulting in a normal or even mildly reduced RI. However, when systemic hypotension falls below the lower limit of autoregulation, a marked reduction in diastolic perfusion pressure slows end‑diastolic flow velocity, leading to a passive increase in RI. Here, the term “passive” is important: under these conditions, elevated RI does not necessarily indicate primary cerebral vasoconstriction, but may instead reflect reduced downstream perfusion pressure secondary to systemic hypoperfusion.

As the disease progresses or when inflammation remains uncontrolled, a sustained or progressive rise in cerebral RI is more indicative of maladaptive pathological mechanisms. These include: (1) severe cerebral microcirculatory disturbances, such as endothelial swelling, microthrombus formation, and leukocyte adhesion causing luminal obstruction [31, 35]. (2) increased intracranial or interstitial pressure due to cerebral edema, compressing the microvascular bed, and (3) profound neurovascular uncoupling [26, 35]. Studies have shown that septic patients—particularly those who develop sepsis‑associated encephalopathy (SAE)—exhibit significantly higher cerebral arterial RI than non‑SAE patients or healthy controls [36]. In this context, an elevated RI no longer represents a compensatory signal but rather clear evidence of microcirculatory failure and cerebral hypoperfusion.

#### Clinical implications related to SAE and interpretative limitations

Abnormally elevated cerebral arterial RI is closely associated with adverse neurological outcomes in sepsis. The clinical spectrum of SAE ranges from mild cognitive impairment to delirium and coma and represents an independent predictor of mortality in sepsis [27, 37, 38]. Higher RI values have been shown to correlate positively with the incidence and severity of SAE as well as with poor mid‑ and long‑term outcomes [39, 40]. From a pathophysiological perspective, an increased RI integrates inflammation‑mediated microvascular injury and impaired cerebral perfusion, providing hemodynamic evidence for the development of SAE and suggesting that resuscitation strategies based solely on traditional MAP targets may be insufficient to ensure adequate cerebral microcirculatory perfusion.

However, several limitations and confounders must be considered when interpreting cerebral RI in clinical practice. First, as noted above, cerebral vessels are extremely sensitive to PaCO₂ [32], mechanical ventilation strategies (e.g., hyperventilation or permissive hypercapnia) can substantially alter RI and obscure sepsis‑related pathological changes. Second, the widespread use of sedative and analgesic agents in the ICU reduces the cerebral metabolic rate of oxygen (CMRO₂) [26] and, via metabolism–flow coupling, may induce cerebral vasoconstriction and an increase in RI. The most critical limitation is the strong blood‑pressure dependence of RI when cerebral autoregulation is impaired. In the absence of effective autoregulation, reductions in systemic blood pressure directly decrease diastolic cerebral blood flow velocity, resulting in an elevated RI. In this setting, an increased RI primarily reflects systemic hypoperfusion rather than a primary increase in cerebrovascular resistance.

In summary, cerebral arterial RI in sepsis is not a static marker of injury but a dynamic window into the interaction among autoregulatory capacity, microcirculatory integrity, and systemic hemodynamics. Its interpretation must therefore be contextualized within the disease stage, global circulatory status, and ventilation/sedation conditions—further underscoring the necessity of serial, rather than single‑time‑point, assessment within the ARISE framework. Recent work on sepsis-associated alterations in cerebral blood flow should also be incorporated here to strengthen the evidence base.

### Renal resistance index: microcirculatory dysfunction and dissociation from global hemodynamics

Among all organ‑related resistance indices, the renal artery RI is arguably the best supported by evidence—and one of the most easily misinterpreted. Its main clinical value lies not in reflecting total renal blood flow, but in revealing sepsis‑related renal microcirculatory dysfunction and its “dissociation” from global systemic hemodynamics [41, 42].

#### Renal vascular architecture and susceptibility to microcirculatory injury

The kidney is a prototypical organ with high perfusion and marked heterogeneity. Under normal physiology, ~ 90% of renal blood flow is distributed to the cortex to support glomerular filtration, whereas medullary blood flow is intrinsically limited yet sustains highly oxygen‑demanding tubular reabsorption [42]. This cortex–medulla mismatch in perfusion makes the kidney particularly vulnerable to disturbances in microcirculatory perfusion.

In sepsis, systemic inflammation, endothelial dysfunction, and abnormal microvascular reactivity can further redistribute intrarenal blood flow: cortical perfusion is relatively preserved, while the medulla is more prone to hypoperfusion and hypoxia. Importantly, this can occur even when microcirculatory variables (e.g., MAP and CO) are maintained or have already been corrected. Thus, sepsis‑associated acute kidney injury(AKI) is, to a large extent, a microcirculation‑driven functional disorder rather than purely a hypoperfusion‑related injury [41, 43]. Renal arterial RI is an ultrasound parameter that is highly sensitive to such changes in microcirculatory resistive load [44].

#### Renal RI in sepsis and septic AKI: dissociation from MAP and prediction of injury

Compared with cerebral blood flow assessment, the use of critical care ultrasound in sepsis‑associated AKI is more mature, with the renal arterial RI being one of the most used and best‑validated parameters [44, 45]. A large body of evidence consistently shows that RI often rises before AKI meets KDIGO diagnostic criteria, and the magnitude of this increase closely correlates with the subsequent severity of renal dysfunction. This suggests that RI primarily reflects early, functional alterations in renal microcirculatory perfusion rather than irreversible structural injury.

In patients with sepsis and septic shock, RI frequently demonstrates a sustained upward trend and is more pronounced in those who ultimately develop AKI. Multiple studies have shown that an elevated RI measured on septic shock independently predicts subsequent AKI and even acute renal failure [46–48]. Conversely, a gradual decline in RI with treatment is usually accompanied by improvement in urine output and recovery of renal function, whereas persistently high RI indicates renal perfusion impairment that may be difficult to reverse [46].

Notably, a marked dissociation often exists between RI and global hemodynamic variables. In some patients with septic shock, increasing MAP from 65 to 75 mmHg with norepinephrine is associated with a significant reduction in RI, suggesting preserved pressure responsiveness of the renal vasculature. In contrast, in patients who have already developed AKI, the RI response to MAP elevation is often markedly blunted. Dewitte et al. reported that in AKI patients, RI correlated more strongly with MAP, age, and the PaO₂/FiO₂ ratio, indicating that renal perfusion at this stage becomes highly dependent on systemic oxygenation and hemodynamic status [45]. This loss of “MAP responsiveness” itself represents an important signal of worsening renal microcirculatory dysfunction.

#### Dynamic interpretation within the ARISE framework

Although renal arterial RI has important predictive value, interpreting a single measurement has notable limitations. RI is an integrated parameter influenced by vascular tone, renal interstitial pressure, venous outflow resistance, and systemic hemodynamic conditions. Interpreting RI in isolation at a single time point—without considering disease stage or therapeutic context—can easily lead to misjudgment.

Within the ARISE framework, the true value of RI lies in serial assessment and in its response pattern to interventions. In early AKI, if an elevated RI decreases after fluid resuscitation or MAP optimization, this often indicates predominantly functional hypoperfusion with good reversibility. In contrast, during progressive or late-stage AKI, persistently high RI with blunted responsiveness to hemodynamic interventions suggests that micro-circulatory injury has become more fixed, and is closely associated with sustained oliguria, need for renal replacement therapy, and poor outcomes. Therefore, renal arterial RI should not be viewed as a static “diagnostic marker” of AKI, but rather as a dynamic window into the renal microcirculatory state and its modifiability. Integrating RI trends with KDIGO staging and treatment response can help differentiate reversible renal hypoperfusion from progressive micro-circulatory damage, thereby providing a more precise basis for individualized hemodynamic management in sepsis-associated AKI. Recent studies combining renal RI with serum biomarkers may further refine this approach and should be cited in an updated reference list.

### Mesenteric resistance index: maladaptive redistribution and the “sacrificial” circulation

During sepsis‑induced blood‑flow redistribution, the mesenteric circulation is arguably the most representative yet least clinically monitored “sacrificial circulation.” Although the gastrointestinal tract receives approximately 25% of CO under physiological conditions [47], its perfusion is often actively downregulated in the early phase of sepsis to preferentially preserve blood flow to vital organs such as the heart and brain [47, 49]. The mesenteric RI provides a unique bedside window for quantifying this process and its downstream consequences.

#### Mesenteric circulation and vulnerability to hypoperfusion

The mesenteric circulation exhibits a pronounced functional–flow coupling. Under physiological conditions, enhanced intestinal motility and the postprandial absorptive state led to marked increases in intestinal wall blood flow, with perfusion of the villi and submucosa reaching several times baseline levels [49, 50]. This finely tuned regulatory mechanism is essential for maintaining adequate mucosal oxygen delivery, meeting metabolic demands, and preserving barrier integrity.

However, this highly dynamic and perfusion‑pressure–dependent regulation also renders the gut particularly vulnerable during sepsis. During systemic stress and circulatory centralization, splanchnic vasoconstriction results in a substantial reduction in mesenteric perfusion. Because the tips of the intestinal villi are intrinsically located in a relatively hypoxic microenvironment, tissue oxygen tension at these sites is the first to decline when perfusion pressure falls, and flow distribution becomes unfavorable. This initiates focal ischemia, followed by mucosal necrosis and disruption of the intestinal barrier [51, 52]. Notably, these processes often occur while conventional macro-circulatory indices—such as MAP, heart rate, and even blood lactate—remain within seemingly acceptable ranges.

#### Mesenteric RI changes in sepsis: adaptive sacrifice versus maladaptive injury

In the early phase of sepsis, an increase in mesenteric RI may, to some extent, be interpreted as a hemodynamic manifestation of adaptive blood‑flow redistribution, whereby splanchnic perfusion is sacrificed to preserve blood flow to central organs [53]. At this stage, mesenteric RI often remains responsive to fluid resuscitation or adjustments in vasoactive therapy, and a transient elevation does not necessarily indicate irreversible injury.

However, a sustained or progressively rising mesenteric RI suggests a transition from this adaptive response to maladaptive injury. Prolonged hypoperfusion, metabolic acidosis, and the combined effects of inflammatory mediators—such as prostaglandins and nitric oxide—can exacerbate intestinal microcirculatory dysfunction, ultimately disrupting the mucosal barrier, increasing the risk of bacterial and endotoxin translocation, and amplifying the systemic inflammatory response [54]. Multiple studies have shown that, during sepsis, mesenteric RI often rises significantly before overt clinical manifestations of gastrointestinal dysfunction become apparent, highlighting its high sensitivity for early microcirculatory injury [55, 56].

#### Clinical relevance and technical challenges

From a clinical perspective, the principal value of mesenteric RI lies in its ability to assess the risk of intestinal ischemia and tolerance to enteral nutrition. Although current guidelines recommend early initiation of enteral nutrition within 24–48 h for ICU patients, approximately 30%–40% of patients with sepsis still develop varying degrees of feeding intolerance [57–59]. Traditional assessment methods—such as bowel sounds, abdominal distension, intra‑abdominal pressure, or lactate levels—are limited in their ability to distinguish transient functional suppression from progressive micro-circulatory injury [60], whereas mesenteric RI allows direct quantification of intestinal perfusion status and its heterogeneity.

At the same time, RI demonstrates rapid dynamic responsiveness to fluid resuscitation, vasoactive agents, and MAP modulation, enabling assessment of whether intestinal perfusion improves in response to therapy [61]. In contrast, persistently elevated RI is closely associated with gastrointestinal failure, prolonged ICU length of stay, and increased mortality risk [62].

Mesenteric RI is not intended to replace traditional macro-circulatory hemodynamic indices, but rather to complement them by addressing their blind spots in the assessment of microcirculation and organ function. Within the ARISE framework, serial monitoring of mesenteric RI may help identify the critical transition point at which blood‑flow redistribution shifts from an “acceptable sacrifice” to overt organ injury, thereby providing physiologically grounded support for enteral nutrition strategies and hemodynamic management in patients with sepsis.

### Peripheral (radial snuffbox artery) resistance index: a window of systemic redistribution

The status of peripheral micro-circulation is closely associated with the prognosis of patients with sepsis, a relationship that is particularly pronounced in septic shock. However, traditional bedside assessment methods mainly focus on the “outcomes” of perfusion (such as skin temperature, mottling, or capillary refill time) and have clear limitations in determining whether micro-circulatory perfusion has truly recovered once macro-circulatory targets have been achieved [63–65]. Growing evidence indicates that even when MAP and CO have been restored to recommended levels, micro-circulatory dysfunction may persist and remain independently associated with poor outcomes, reflecting a typical phenomenon of macro–micro-circulatory dissociation [66–68]. Peripheral arterial resistance indices, especially the snuffbox RI, provide a simple and reproducible bedside window for identifying this dissociation.

#### Rationale for peripheral arterial RI monitoring

From a physiological perspective, peripheral arterioles represent the primary effector site for regulating systemic vascular tone [69]. In sepsis, neurohumoral activation, inflammation‑mediated endothelial dysfunction, and impaired vasoreactivity emerge early and are particularly pronounced within the peripheral vascular bed [70]. Accordingly, peripheral vascular indices may serve as surrogate markers of global vascular tone and vascular compliance, rather than reflecting merely local hemodynamic conditions. From a practical perspective, peripheral arterial RI offer several advantages [71]. The radial snuffbox artery, a superficial branch of the radial artery coursing through the anatomical snuffbox on the dorsum of the hand, has a consistent anatomical location and excellent sonographic accessibility [71]. Even in critically ill patients with marked hemodynamic instability, reproducible Doppler waveforms can be obtained with minimal procedural burden. These features make RI measurement at this site one of the few bedside, repeatable, and quantitative approaches for monitoring peripheral vascular status in the ICU.

#### Snuffbox artery RI and systemic hemodynamic adaptation

Compared with largely subjective peripheral perfusion indices, the snuffbox RI provides a quantitative and repeatable approach to assessing peripheral vascular regulation. Clinical studies have shown that an increased snuffbox artery RI is closely associated with impaired tissue perfusion [72]. In critically ill patients with severe infection, snuffbox artery RI outperformed the perfusion index (PI) in predicting elevated blood lactate levels, whereas PI appeared to be more reflective of impaired lactate clearance capacity [73]. This distinction suggests that snuffbox artery RI more directly captures abnormalities in peripheral vascular resistance and microcirculatory hypoperfusion per se, rather than downstream metabolic consequences.

In the context of parallel, multi-organ RI assessment, peripheral RI also serves as an important reference comparator. When cerebral or renal RI improves while snuffbox artery RI remains elevated, this pattern often indicates a redistribution state in which peripheral perfusion is sacrificed to preserve perfusion of vital organs. Conversely, if peripheral RI decreases with treatment but central-organ RI fails to improve in parallel, it may imply an organ-specific pathological process dominated by local micro-circulatory dysfunction. Thus, snuffbox artery RI provides essential systemic context for interpreting changes in resistance indices of core organs.

#### Role of within the ARISE framework

Within the ARISE framework, peripheral RI is not intended to assess the function of a specific organ; it serves as a reference context for systemic blood-flow redistribution. Its principal value lies in helping distinguish acceptable adaptive redistribution from maladaptive, sustained sacrifice of peripheral perfusion.

Although a single snuffbox artery RI measurement has inherent limitations, its longitudinal trajectory and treatment-response patterns are highly informative. After fluid resuscitation or adjustment of vasoactive agents, a gradual decline in snuffbox artery RI generally suggests recovery of peripheral vascular tone and improvement in micro-circulatory perfusion. By contrast, persistently elevated snuffbox artery RI despite achievement of macro-circulatory targets indicates ongoing macro–micro circulatory uncoupling and may warrant re-evaluation of the resuscitation strategy and hemodynamic goals.

Accordingly, the radial snuffbox arterial RI provides a low-cost, readily obtainable, and physiologically grounded integrative node for multi-organ RI assessment within the ARISE framework, facilitating interpretation of organ-specific signals within the broader context of systemic blood-flow redistribution.

### Integrating cross-organ RI within the ARISE framework

RI derived from different arterial vascular beds should not be interpreted as isolated measures that merely reflect the status of a single organ. Rather, when considered collectively, these RIs constitute a set of complementary signals that can capture the dynamic pattern of blood-flow redistribution characteristic of sepsis. Within the ARISE framework, RI serves as a functional interface linking systemic hemodynamics, regional autoregulatory capacity, and microcirculatory integrity, thereby shifting perfusion assessment from single-target, goal-directed resuscitation toward a systems-level, integrative evaluation. Taken together, these four RI measurements delineate a continuum of septic perfusion responses—from preserved autoregulation, through a fragile micro-circulatory phase, to intentional yet ultimately maladaptive “sacrifice” of blood flow. Importantly, the ARISE framework conceptualizes RI not as a static marker of injury, but as a dynamic indicator of adaptive reserve, redistribution strategy, and the reversibility of micro-circulatory dysfunction. Serial assessment is therefore essential. Divergent trajectories across vascular beds may indicate ongoing redistribution at the expense of vulnerable regions, whereas concordant improvement more likely reflects global recovery of micro-circulatory function.

Potential modifiers and confounders should be carefully considered when interpreting ARISE-derived organ-specific RI. RI is influenced not only by local arterial resistance but also by vascular compliance, pulse pressure, venous outflow, interstitial pressure, and systemic hemodynamic conditions [74]. Therefore, in elderly patients or those with multiple comorbidities, pre-existing organ dysfunction, or macrovascular/microvascular disease, baseline RI values and their temporal responses may be substantially altered [75, 76]. Chronic kidney disease may increase renal RI through renal fibrosis and arteriolosclerosis [77]. Diabetes and peripheral vascular disease may modify peripheral or organ-specific RI through microangiopathy, endothelial dysfunction, and reduced vascular compliance [74]. Prior abdominal surgery, pancreatitis, or intra-abdominal infection may complicate mesenteric RI interpretation through anatomical distortion, local edema, altered splanchnic flow, or increased intra-abdominal pressure [78]. Right heart failure may elevate RI by increasing venous pressure and impairing diastolic forward flow [79, 80]. Treatment-related factors, including vasopressor dose, fluid balance, mechanical ventilation settings, positive end-expiratory pressure, sedation depth, and venous congestion, should also be documented and incorporated into interpretation [10, 81].

Serial assessment may reduce inter-individual bias by emphasizing within-patient trajectories rather than fixed universal cutoffs. Nevertheless, chronic diseases may shift baseline RI and alter the vascular response to acute resuscitation. Thus, ARISE results should be interpreted according to the direction, magnitude, and concordance of RI changes across vascular beds, together with clinical history, baseline organ function, infection source, vasoactive support, ventilatory conditions, and other perfusion markers. ARISE may be more reliable when advanced chronic vascular disease or end-stage organ failure is absent and when the infection source does not directly involve the vascular bed being assessed. Conversely, interpretation should be more cautious in abdominal sepsis, severe pancreatitis, intra-abdominal hypertension, urinary-source sepsis, prior complex abdominal surgery, or significant right heart failure. These considerations reinforce that ARISE should serve as an adjunctive dynamic tool for assessing heterogeneity of organ perfusion, rather than a standalone diagnostic criterion independent of clinical context.

## Clinical implications of the ARISE framework for resuscitation strategies, vasoactive drug selection, and monitoring pathways

In the resuscitation of sepsis, current mainstream strategies remain largely centered on achieving macro-circulatory targets such as MAP and CO. However, this “target-achievement–based” approach has limited ability to identify inter-organ perfusion redistribution and its potential physiological costs. By integrating the dynamic behavior of multi-organ arterial RI, the ARISE framework introduces an additional decision-making dimension that enables more refined adjustment of resuscitation strategies.

### Implications for resuscitation strategy

At the level of resuscitation strategy, ARISE supports a shift from single global targets toward organ-oriented resuscitation. When MAP has reached recommended thresholds, but renal or mesenteric RI remains elevated, which suggests the presence of macro–microcirculatory uncoupling; in such circumstances, further increases in arterial pressure alone may fail to improve—and may even worsen—perfusion of vulnerable organs. Conversely, when peripheral RI is markedly elevated while cerebral RI remains stable, the pattern is more consistent with redistribution dominated by systemic vasoconstriction, indicating that resuscitation strategies should carefully balance vascular tone against regional perfusion. By recognizing composite patterns across organ-specific RI trajectories, ARISE helps determine whether resuscitation has truly achieved “perfusion improvement,” rather than merely numerical target attainment.

### Implications for vasoactive drug selection

With respect to vasoactive therapy, ARISE provides a physiological basis for evaluating drug effects at the organ level. Snuffbox RI serves as a real-time window into systemic vascular tone and the overall effects of vasoactive agents; cerebral RI reflects whether central perfusion is compromised by excessive vasoconstriction; and renal and mesenteric RIs reveal the sensitivity of the micro-circulation to different pharmacological regimens. Through serial comparison of RI response patterns across vascular beds, ARISE facilitates discrimination between states of “effective pressure augmentation with impaired perfusion” and those in which vascular tone and perfusion are relatively balanced. This, in turn, informs dose titration of vasoconstrictors such as norepinephrine and supports decisions regarding the addition or transition to vasodilatory or inotropic agents, rather than relying solely on changes in MAP.

### Implications for monitoring pathways

From a monitoring perspective, ARISE offers a bedside-implementable, integrative, and dynamic assessment framework. Using peripheral RI as a reference context for systemic vascular status, combined with serial monitoring of cerebral, renal, and mesenteric RIs, a continuous monitoring pathway can be constructed that spans from global circulatory regulation to organ-specific perfusion. Divergent RI trends across vascular beds suggest ongoing redistribution that may occur at the expense of vulnerable organs, whereas concordant improvement across multiple RIs more likely reflects global recovery of micro-circulatory function. This trend-based, rather than single-point, monitoring approach positions ARISE as a closed-loop tool linking assessment, intervention, and reassessment.

In practice, a stepwise implementation strategy may improve bedside usability: (1) establish systemic hemodynamic context using standard monitoring and focused ultrasound; (2) obtain baseline RI measurements in the most feasible vascular beds; (3) repeat RI assessment after major interventions such as fluid administration, vasopressor titration, or ventilatory adjustment; and (4) interpret changes according to trajectory concordance or divergence rather than absolute thresholds alone. This process should ideally be integrated with lung ultrasound, venous congestion assessment, and routine clinical markers such as lactate, urine output, and mental status. At the same time, a full ARISE evaluation may be time-consuming and technically demanding, especially when combined with standard POCUS components such as cardiac, lung, inferior vena cava, or venous congestion assessment (Table 1). Its feasibility is therefore likely to be greatest in ICU settings with trained operators and may be more limited in emergency departments, high-dependency units, or general wards.

**Table 1:** Proposed practical framework for serial multi-territory resistance index assessment within the ARISE model

Territory/organ	Suggested target vessel(s)	Ultrasound technique	Minimum technical requirements	Suggested timing for reassessment	Time*	Major confounders and limitations
Cerebral circulation	Middle cerebral artery (MCA)	Transcranial Doppler or transcranial color-coded duplex with pulsed-wave Doppler sampling	Adequate temporal acoustic window, operator familiarity with intracranial vascular anatomy; stable insonation from a consistent window; acquisition of reproducible waveforms	Baseline assessment after initial hemodynamic evaluation; repeat after major therapeutic interventions, including fluid administration, vasopressor adjustment, or significant ventilatory changes	3–5 min	Poor temporal window, advanced age, agitation, head dressings/devices, PaCO2 changes, vasoactive drugs, operator dependency
Renal circulation	Segmental or interlobar renal arteries (preferably bilateral)	Color Doppler localization followed by pulsed-wave Doppler using curvilinear or phased-array probe	Identification of comparable intrarenal sampling sites; minimal transducer compression; acquisition of at least 3 reproducible waveforms; averaging of repeated measurements when possible	Early baseline during resuscitation; repeat after fluid challenge, vasopressor modification, worsening kidney function, or decongestive interventions	5–8 min	Obesity, bowel gas, mechanical ventilation, venous congestion, chronic kidney disease, elevated intra-abdominal pressure, poor reproducibility in difficult windows
Superior mesenteric artery (SMA)	Proximal SMA	Upper abdominal color Doppler localization followed by pulsed-wave Doppler	Reliable identification of the SMA; angle optimization; stable respiratory conditions when possible; reproducible sampling from the same proximal segment; acquisition of reproducible waveforms	Baseline if technically feasible; repeat after major hemodynamic interventions or when persistent splanchnic hypoperfusion is suspected	4–7 min	Bowel gas, obesity, prior abdominal surgery, pancreatitis, ileus, abdominal distension, feeding status, respiratory motion, marked operator dependency
Peripheral circulation	Distal radial artery in the anatomical snuffbox	Linear probe with color Doppler localization followed by pulsed-wave Doppler	Consistent use of the same side and same arterial segment for serial measurements; minimal probe pressure; stable hand positioning; acquisition of at least three reproducible waveforms under comparable local perfusion conditions	Baseline and during serial reassessment of peripheral perfusion, particularly during vasopressor titration or evolving shock	2–4 min	Small vessel caliber, local vasospasm, probe pressure sensitivity, hand position variability, skin temperature, vasopressor use, peripheral arterial disease, operator dependency, limited validation as a standardized RI target in septic shock
Integrated multi-organ assessment	Combined cerebral, renal, SMA, and peripheral RI evaluation, ideally interpreted alongside cardiac and lung ultrasound	Sequential focused multi-organ Doppler assessment at the bedside	Advanced critical care ultrasonography skills; consistency of serial measurements; integration with hemodynamic status, vasoactive support, and ventilator settings	Initial comprehensive examination early in the resuscitation course, followed by targeted reassessment after clinically meaningful interventions or deterioration	15–25 min	Time burden, incomplete acoustic windows, operator dependency, limited standardization, uncertain inter-observer reproducibility

This table summarizes a pragmatic, non-standardized approach to serial multi-territory RI assessment. At present, no universally validated protocol defines the optimal vascular targets, acquisition sequence, or reassessment intervals for ARISE-guided monitoring

*ARISE* arterial resistance index-based integrated serial evaluation, *MCA* middle cerebral artery, *RI* resistance index, *SMA* superior mesenteric artery

*Approximate acquisition time refers to focused image acquisition under favorable conditions by an operator experienced in critical care ultrasonography; total examination time may be substantially longer in unstable or technically difficult patients

**Fig. 2:**
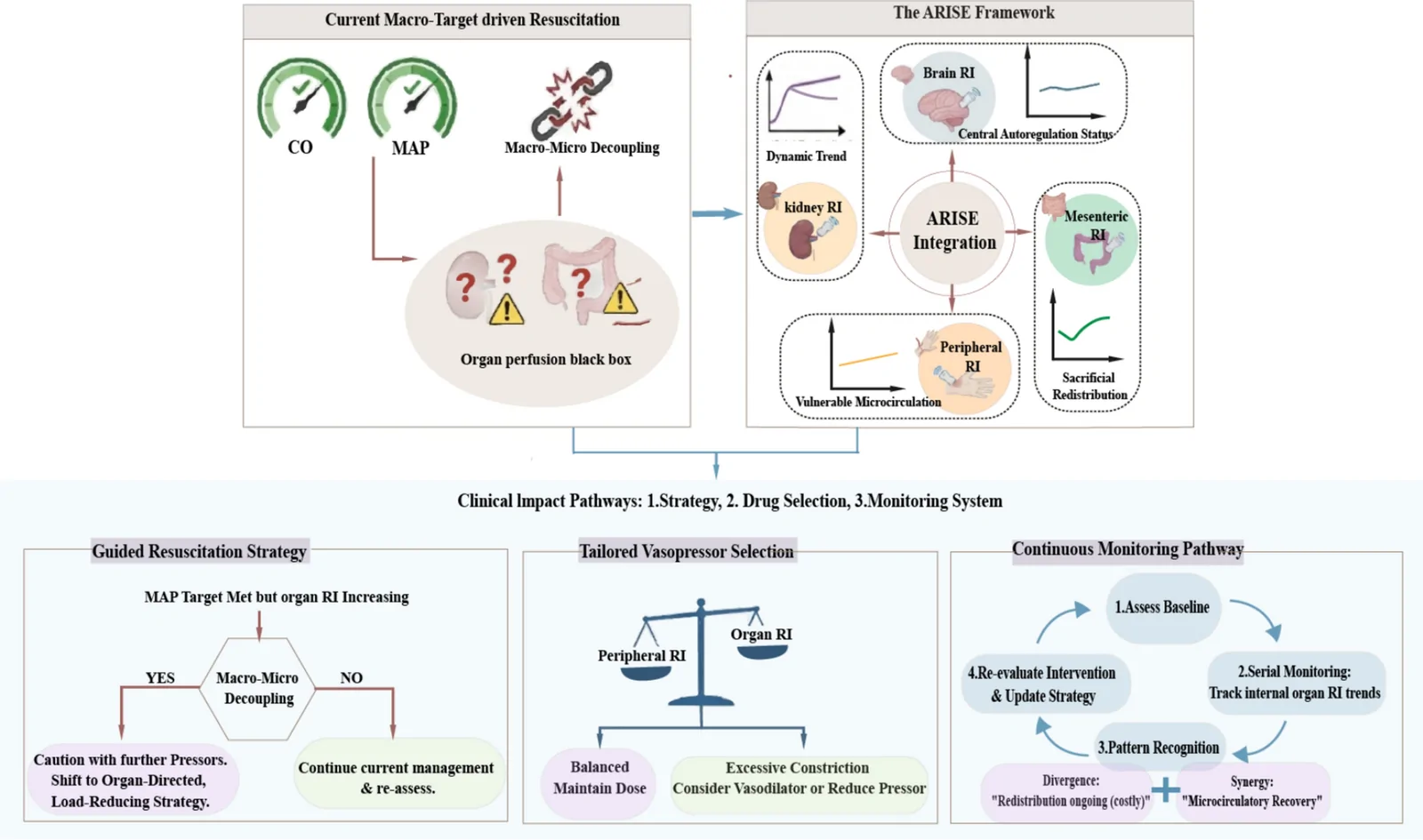
The ARISE framework bridging multi-organ hemodynamic signals of precision medicine–guided therapy

In summary, the ARISE framework introduces dynamic integration of multi-organ RIs into the hemodynamic management of sepsis, providing actionable physiological guidance for optimizing resuscitation strategies, individualizing vasoactive drug selection, and structuring bedside monitoring pathways. By identifying patterns of blood-flow redistribution rather than focusing exclusively on macro-circulatory targets, ARISE has the potential to advance sepsis management from a “pressure- and flow-oriented” paradigm toward a more precise resuscitation model that is informed by perfusion and redistribution awareness, as shown in Fig. 2.

Conventional “macro-target-driven” resuscitation primarily aims to achieve global hemodynamic targets such as mean arterial pressure (MAP) and cardiac output (CO). However, in the presence of macro–microcirculatory uncoupling, true organ perfusion may remain a “black box,” making it difficult to promptly detect impaired perfusion or blood-flow redistribution in key organs such as the kidney and gut. The ARISE framework enables a dynamic, integrative assessment by serial bedside Doppler measurements of multi-organ arterial resistive indices (RI) (e.g., cerebral, renal, mesenteric, and peripheral RI). This approach can inform clinical management in three domains: (1) resuscitation strategy—when MAP is at target yet renal/mesenteric RI increases, uncoupling should be suspected; further escalation of vasopressors should be undertaken cautiously, with a shift toward organ-oriented strategies that reduce hemodynamic load and optimize organ perfusion; (2) selection and titration of vasoactive agents—peripheral RI can serve as a surrogate of vascular tone, whereas organ RI reflects perfusion; therapy should balance inadequate vascular tone against excessive vasoconstriction (with consideration of adding inotropes, de-escalating vasopressors, or introducing vasodilators when appropriate); (3) longitudinal monitoring—establish a baseline, track serial changes in internal-organ RI, identify physiological states (divergent trajectories indicating redistribution; concordant decreases suggesting microcirculatory recovery), and iteratively reassess and update the therapeutic plan accordingly.

Although the ARISE framework provides a physiologically grounded and organ-oriented approach to sepsis resuscitation, it remains conceptual and requires validation before clinical implementation. Existing evidence supports several components of the framework, including the limitations of global hemodynamic targets in capturing regional perfusion heterogeneity, the association of Doppler-derived RI with organ-specific hemodynamics and outcomes, and the value of serial within-patient assessment over isolated measurements. However, key uncertainties remain regarding the optimal vascular beds, standardized acquisition protocols, measurement frequency, clinically meaningful RI changes, and the prognostic significance of cross-organ RI divergence. The influence of comorbidities, infection source, vasopressor therapy, mechanical ventilation, intra-abdominal pressure, and venous congestion also needs systematic evaluation. Future research should proceed from methodological studies assessing reproducibility and standardization, to prospective observational studies linking ARISE trajectories with organ dysfunction and outcomes, and ultimately to interventional trials testing whether ARISE-guided resuscitation improves patient-centered outcomes compared with standard care. Until then, ARISE should be considered a hypothesis-generating and adjunctive monitoring framework rather than a validated resuscitation protocol.

## Conclusion

Sepsis is a syndrome characterized by marked temporal evolution and profound heterogeneity in organ involvement. Its circulatory derangements are not simply a matter of “low blood pressure” or “insufficient flow,” but rather reflect a dynamic process of blood-flow redistribution and perfusion imbalance as the disease progresses. The arterial resistive index (RI), an ultrasound-derived parameter that captures organ vascular resistance/compliance and relates to organ perfusion, is clinically actionable and constitutes the physiological basis of the ARISE framework. Bedside Doppler-based RI assessment provides a feasible, repeatable, near–real-time approach to monitoring organ perfusion. The strength of ARISE does not lie in single, single-organ “point” measurements; instead, it integrates RI signals across multiple organs and emphasizes their trajectories over time. This offers a structured lens through which to characterize sepsis-associated circulatory abnormalities, shifting the evaluative focus from static attainment of global hemodynamic targets (e.g., mean arterial pressure [MAP]) to the identification, comparison, and interpretation of inter-organ perfusion redistribution patterns.

By focusing on which organs develop rising resistance and impaired perfusion, when these changes occur, whether trajectories are concordant or divergent across organs, and how they relate to clinical outcomes and responses to interventions, ARISE may more faithfully reflect true organ perfusion status. It may provide earlier, more forward-looking dynamic signals of impending organ dysfunction, facilitate early identification of vulnerable organs, refine interpretation of responses to fluid resuscitation and vasoactive therapies, and support more individualized, physiology-guided management strategies in sepsis.

However, ARISE should currently be viewed as a conceptual framework rather than a fully validated clinical protocol. Important evidence gaps remain regarding standardized acquisition, inter-operator reproducibility, organ-specific confounders, optimal reassessment intervals, and—most importantly—whether ARISE-guided management improves patient-centered outcomes such as organ failure, renal replacement therapy use, ICU length of stay, or mortality. Future studies should therefore include prospective observational validation, standardized feasibility assessments, and interventional trials comparing ARISE-informed resuscitation with conventional hemodynamic strategies.

## Data Availability

Data sharing is not applicable to this article as no new data were created or analyzed in this study.
